# The relationship between cognitive function and inflammatory-nutritional status in schizophrenia: cognitive impairment is inversely related to PNI and CALLY indices

**DOI:** 10.1186/s12888-026-08021-0

**Published:** 2026-03-27

**Authors:** Okan Imre, Aysegul Kirkas

**Affiliations:** Department of Psychiatry, Karamanoglu Mehmetbey University Faculty of Medicine, Karaman, Turkey

**Keywords:** Schizophrenia, Cognitive impairment, PNI, CALLY index, Inflammation, Nutrition

## Abstract

**Background:**

Cognitive impairment in patients with schizophrenia is one of the symptoms most closely associated with functional loss throughout the disease course. There is increasing evidence suggesting that inflammation and nutritional status may contribute to impaired cognitive functions. The C-reactive protein/albumin ratio (CRP/ALB), prognostic nutritional index (PNI), and C-reactive protein–albumin–lymphocyte index (CALLY index) are easily calculated composite biomarkers reflecting nutritional status and immune-inflammatory processes. This study investigated the associations of the CRP/ALB, PNI, and CALLY indices with cognitive impairment in patients with schizophrenia.

**Methods:**

A total of 131 individuals (88 schizophrenia patients and 43 healthy controls) were included. Cognitive assessment was performed via the Montreal Cognitive Assessment Scale (MoCA). On the basis of the MoCA, 26 patients had cognitive impairment (SCH-CI), and 62 were classified as schizophrenia patients with preserved cognitive function (SCH-R), and 43 constituted the healthy control (HC) group. Serum ALB, lymphocyte, and CRP levels were first measured. The CRP/ALB ratio, PNI, and CALLY indices were then calculated on the basis of these parameters. Group differences were assessed via statistical analyses.

**Results:**

The CRP/ALB ratio was significantly higher in both schizophrenia groups than in the HC group (p2 < 0.001; p3 < 0.001), whereas no significant difference was observed between the SCH-CI and SCH-R groups (p1 = 1.000). The CALLY index was significantly lower in both schizophrenia groups than in the HC group (p2 < 0.001; p3 < 0.001), with no significant unadjusted difference between SCH-CI and SCH-R (p1 = 0.200). PNI values were significantly lower in the SCH-CI group than in both the SCH-R (p1 = 0.008) and HC groups (p2 = 0.013), whereas no significant difference was observed between the SCH-R and HC groups (p3 = 1.000). In multivariable logistic regression analyses adjusted for age, sex, body mass index, smoking status, education year, depot antipsychotic use, and treatment duration, the CALLY index remained independently associated with cognitive impairment (OR = 0.112, 95% CI: 0.013–0.943, *p* = 0.044), whereas the association for PNI was attenuated to borderline significance (OR = 0.895, 95% CI: 0.800–1.001, *p* = 0.052). The CRP/ALB ratio was not independently associated with cognitive impairment (OR = 13.363, 95% CI: 0.147–1212.867, *p* = 0.260).

**Conclusions:**

Cognitive impairment in schizophrenia may be associated with disturbances in inflammatory and immunonutritional status. Among the evaluated biomarkers, the CALLY index showed the most robust independent association with cognitive impairment after adjustment for relevant covariates, whereas the association for PNI was weaker. Although these markers are inexpensive and easily obtainable from routine laboratory parameters, their modest discriminatory performance suggests that they may be more useful as supportive or risk-stratification markers than as standalone diagnostic tools. Larger prospective studies are needed to validate their clinical utility.

## Background

Schizophrenia is a chronic psychiatric disorder characterized by lifelong impairment and substantial disturbances in cognitive, social, and occupational functioning [1]. Classically, it is defined by positive symptoms (e.g., delusions, hallucinations), negative symptoms (e.g., social withdrawal, anhedonia, loss of interest and motivation), and cognitive deficits affecting domains such as orientation, concentration, memory, language, attention, and executive functioning [2]. If untreated, the fluctuating course of positive, negative, and cognitive symptoms can result in significant biopsychosocial consequences, including physical and mental health problems, social dysfunction, loss of productivity, and increased economic burden [3]. Although current pharmacological treatments are largely effective in managing positive and negative symptoms, there remains no established therapeutic intervention for cognitive deficits, likely owing to the limited understanding of their pathophysiological underpinnings [4]. Recent research has increasingly emphasized that cognitive impairment represents the most persistent and functionally disabling dimension of schizophrenia [5, 6].

The pathophysiology of cognitive dysfunction in schizophrenia is multifactorial and involves genetic susceptibility, neurotransmitter dysregulation, and structural and functional brain alterations. However, accumulating evidence suggests that systemic inflammation and nutritional status may also contribute to this process [7–9]. Elevated inflammatory cytokines, reduced serum ALB, and decreased lymphocyte counts have been associated with both schizophrenia and cognitive impairment [10, 11].

The CRP/ALB ratio, prognostic nutritional index (PNI), and C-reactive protein–albumin–lymphocyte (CALLY) index are composite biomarkers that reflect both inflammatory and nutritional status and are easily calculated from routine laboratory tests. The CRP/ALB ratio combines C-reactive protein as a marker of inflammation with albumin as an indicator of nutritional state [12]. The PNI integrates the serum ALB concentration and lymphocyte count to reflect nutritional and immune function [13]. The CALLY index incorporates CRP, albumin, and lymphocyte levels and is considered a balanced marker of systemic inflammation and nutritional status [14]. While these indices have been investigated in other medical conditions, such as oncology and cardiovascular diseases, their application in psychiatric disorders—particularly schizophrenia with cognitive impairment—remains limited.

Although inflammatory and immunonutritional markers including the CRP/ALB ratio and PNI have been studied in schizophrenia, their association with cognitive impairment is poorly characterized, and the CALLY index has not yet been explored in this clinical context. Accordingly, the present study aims to address this gap by comparatively evaluating the CRP/ALB ratio, PNI, and CALLY index in schizophrenia patients with and without cognitive impairment, in order to determine their potential discriminatory value and clinical relevance.

## Materials and methods

### Ethical approval and participants

This case–control study included patients diagnosed with schizophrenia who were receiving follow-up and treatment at the Psychiatry Clinic of Karaman Training and Research Hospital. All participants were between 18 and 65 years of age. The diagnosis of schizophrenia was made by specialist psychiatrists through a comprehensive clinical evaluation based on the Diagnostic and Statistical Manual of Mental Disorders, Fifth Edition, Text Revision (DSM-5-TR). Diagnostic procedures included structured clinical interviews, detailed psychiatric examinations, and review of medical records. All patients were in the chronic disease phase at the time of enrollment and were receiving ongoing antipsychotic treatment. Ethical approval was obtained from the Clinical Research Ethics Committee of Karamanoglu Mehmetbey University Faculty of Medicine (approval number 23-2025/17, dated 10.09.2025). The study was conducted in accordance with the principles of the Declaration of Helsinki, as revised in 2013. Written informed consent was obtained from all participants.

**The exclusion criteria included** uncontrolled diabetes mellitus, hypertension, malnutrition, active infection, cardiovascular disease, inflammatory disorders, neurological diseases, extreme biochemical values, hearing or speech impairments, inability to cooperate with testing, lack of informed consent, and the use of medications other than psychiatric drugs. Substance users were excluded from both the patient and healthy control (HC) groups, as substance use is a confounding factor in cognitive impairment.

Prior to data collection, an a priori power analysis was conducted using G*Power version 3.1 for a fixed-effects one-way ANOVA comparing three independent groups (SCH-CI, SCH-R, and HC). The effect size was set at f = 0.30, based on the magnitude of between-group differences in the CRP/albumin ratio reported by Balcioglu et al. (2020). Assuming a two-sided α error of 0.05 and a desired power (1–β) of 0.80, the required total sample size was estimated to be 108 participants. Our final sample of 131 participants (26 SCH-CI, 62 SCH-R, and 43 HC) exceeded this requirement, indicating adequate statistical power for the primary between-group comparisons [15]. A total of 88 schizophrenia patients who met the study criteria were included. Additionally, 43 healthy controls (4 females and 39 males) without a history of psychiatric illness or exclusion criteria were recruited as the HC group.

Sociodemographic data were recorded for all schizophrenia patients. Cognitive function was assessed by two experienced clinical psychologists via the Montreal Cognitive Assessment Scale (MoCA). On the basis of the MoCA scores, 26 patients were classified as having cognitive impairment (SCH-CI), whereas 62 were categorized as schizophrenia patients with preserved cognitive function (SCH-R). Blood samples were collected following an overnight fast of 8 h. CRP, albumin, and lymphocyte levels were analyzed from blood samples collected during the routine clinical examinations of the participants. For serum separation, blood samples were allowed to clot for 30 min and subsequently centrifuged at 1800×g for 10 min at 8 °C. Supernatants were immediately transferred to microtubes and stored at -80 °C until biochemical analysis. CRP (0–5 mg/L) and albumin (3.5–5.5 g/dl) levels were measured using the Advia-Centaur XP automated analyzer (Siemens AG, Munich, Germany) with parameter-specific routine kits. The lymphocyte parameter (0.8-4 × 10³/µL) was determined using the Mindray BC 6800 automated hematology analyzer (Mindray, Shenzhen, China). Serum albumin (g/dL), lymphocyte count (/mm³), and C-reactive protein (CRP, mg/L) levels were measured. From these parameters, the prognostic nutritional index (PNI) and CALLY index were calculated. Biochemical data, inflammatory markers, and nutritional indices were compared among the schizophrenia subgroups and HCs.

### Index calculations

PNI = 10×albumin (g/dL) + 0.005×lymphocyte count (/mm3)

CALLY index= [albumin (g/dL) × lymphocyte count (/mm³)] / [CRP (mg/L) × 10⁴]

Albumin, lymphocyte count, and CRP were expressed as g/dL, cells/mm³, and mg/L, respectively [14].

### Montreal cognitive assessment scale (MoCA)

The MoCA was developed by Dr. Ziad Nasreddine in Montreal, Canada, for the detection of mild cognitive impairment (MCI). Short-term memory, attention, working memory, and executive function were assessed with the MoCA test. The evaluation consists of a 30-point test that can be administered in 10–15 min. Cognitive function was considered normal in individuals with a score of 26 or above. A score of 25 or less on the MoCA indicates cognitive impairment. The MoCA is an appropriate tool for the assessment of cognitive function in patients with schizophrenia and has been shown to be as effective as other commonly used cognitive tests in this population [16–18].

### Statistical analysis

Data analyses were performed via SPSS for Windows, version 25.0 (IBM Corp., Armonk, NY, USA). The normality of continuous variables was evaluated via the Kolmogorov–Smirnov test, Q–Q plots, skewness, and kurtosis values. Normally distributed variables are presented as the means ± standard deviations, whereas nonnormally distributed variables are reported as medians (Q1–Q3). For between-group comparisons, the independent samples t test or one-way ANOVA was applied for normally distributed variables, whereas the Kruskal–Wallis test was used for nonnormally distributed variables. When overall group differences were statistically significant, post hoc pairwise comparisons were conducted using the Bonferroni correction for ANOVA, Tamhane’s T2 test in the presence of unequal variances, and the Dunn–Bonferroni procedure following the Kruskal–Wallis test. Categorical variables are expressed as frequencies and percentages and were compared via the chi-square test.

Correlations between variables were examined via Pearson or Spearman correlation coefficients, depending on distributional characteristics. The correlation strength was interpreted as follows: weak = 0.01–0.49, moderate = 0.50–0.69, and strong = 0.70–1.00. To assess discriminatory performance, receiver operating characteristic (ROC) curve analysis was conducted, and the area under the curve (AUC) values with 95% confidence intervals were reported. The optimal cut-off points, sensitivity, specificity, and likelihood ratios were determined, with the highest likelihood ratio considered for cut-off selection.

To account for potential confounding, multivariable binary logistic regression analyses were additionally performed, in which cognitive impairment (SCH-CI vs. SCH-R) was entered as the dependent variable and each inflammatory–nutritional index (PNI, CALLY index, and CRP/albumin ratio) was evaluated as the main predictor, while adjusting for age, sex, body mass index (BMI), smoking status, education year and depot antipsychotic use. Covariate selection was based on a priori clinical relevance and previous literature rather than automated procedures. Model fit was assessed via the Hosmer–Lemeshow test. Adjusted effect estimates were reported as odds ratios (ORs) with 95% confidence intervals.

For all analysis results, a significance level of *p* < 0.05 was accepted.

## Results

A total of 131 individuals were included in the study. Among them, 19.8% (*n* = 26) were classified into the schizophrenia–cognitive impairment group (SCH-CI), 47.3% (*n* = 62) into the schizophrenia with preserved cognitive function group (SCH-R), and 32.8% (*n* = 43) into the healthy control (HC) group. No statistically significant difference was observed among the groups regarding sex distribution (*p* = 0.079) or age (*p* = 0.460) (Table 1).

**Table 1:** Gender and age distribution of the study groups

	SCH-CI (*n* = 26)	SCH-R (*n* = 62)	HC(*n* = 43)	
	Mean ± Std. Dev Median(Q1-Q3)	Mean ± Std. Dev Median(Q1-Q3)	Mean ± Std. Dev Median(Q1-Q3)	*p*
Gender -Male (n(%))	18(69.2%)	50(80.6%)	39 (90.7%)	0.079^χ2^
Age	49.81 ± 10.04	46.74 ± 8.37	47.91 ± 13.33	0.460*

* ANOVA, ^χ2^ chi-square test;

Body mass index (BMI) was also comparable, with no significant difference between the groups (*p* = 0.315). Educational duration also differed significantly between the groups, with the SCH-CI group having fewer years of education (*p* = 0.003). There was no significant difference in illness duration between the groups (*p* = 0.057) No significant differences were found between the SCH-CI and SCH-R groups regarding marital status (*p* = 0.960), smoking (50.0% vs. 64.5%, *p* = 0.204), comorbid medical conditions (30.8% vs. 35.5%, *p* = 0.670), or psychiatric comorbidities (*p* = 0.938). The rate of depot antipsychotic use was 34.6% in the SCH-CI group and 46.8% in the SCH-R group, with no significant intergroup difference (*p* = 0.293). Additionally, MS use was reported in 23.1% of SCH-CI patients and 22.6% of SCH-R patients, and this difference was likewise not statistically significant (*p* = 0.960) (Table 2).

**Table 2:** Comparison of sociodemographic and clinical characteristics between schizophrenia subgroups

		SCH-CI (*n* = 26) *n*(%)-Mean± Std. Dev.	SCH-R (*n* = 62) *n*(%)-Mean± Std. Dev.	*p*
BMI (kg/m²)		28.904 ± 5.98	30.326 ± 6.03	0.315*
Education (year)		6.15 ± 3.16	8.32 ± 2.95	0.003*
Illness duration		24.92 ± 11.41	20.56 ± 8.88	0.057*
Married	Yes	6 (23.1%)	14 (22.6%)	0.960 ^χ2^
	No	20 (76.9%)	48 (77.4%)	
Cigarette	Yes	13 (50.0%)	40 (64.5%)	0.204 ^χ2^
	No	13 (50.0%)	22 (35.5%)	
Additional disease	Yes	8 (30.8%)	22 (35.5%)	0.670 ^χ2^
	No	18 (69.2%)	40 (64.5%)	
Psychiatric disorder	Yes	9 (34.6%)	22 (35.5%)	0.938 ^χ2^
	No	17 (65.4%)	40 (64.5%)	
Depot antipsychotic	Yes	9 (34.6%)	29 (46.8%)	0.293 ^χ2^
	No	17 (65.4%)	33 (53.2%)	
Mood stabilizer	Yes	6 (23.1%)	14 (22.6%)	0.960 ^χ2^
	No	20 (76.9%)	48 (77.4%)	

*Independent samples test, ^χ2^Chi-square tests

Lymphocyte counts differed significantly among the groups (*p* = 0.043). Pairwise comparisons demonstrated that SCH-R patients had higher lymphocyte counts than SCH-CI patients did (p1 = 0.037), while no significant differences were found between SCH-CI patients and HCs (p2 = 0.252) or between SCH-R patients and HCs (p3 = 1.000).

The serum ALB levels also differed significantly between the groups (*p* = 0.023), with the SCH-CI group exhibiting significantly lower levels than the HC group (p2 = 0.019). However, no significant differences were identified between SCH-CI and SCH-R (p1 = 0.142) or SCH-R and HC (p3 = 0.804).

CRP levels were highly significantly different between the two groups (*p* < 0.001). Pairwise comparisons indicated that both patient groups had higher CRP levels than did the HC group (p2 < 0.001; p3 < 0.001), whereas no significant difference was detected between the SCH-CI and SCH-R groups (p1 = 1.000).

Similarly, the CRP/ALB ratio differed significantly across groups (*p* < 0.001) and was greater in both schizophrenia groups than in the HCs (p2 < 0.001; p3 < 0.001), with no significant difference between SCH-CI and SCH-R (p1 = 1.000).

A significant group difference was also noted in the CALLY index (*p* < 0.001), with HC participants exhibiting higher values than both patient groups did (p2 < 0.001; p3 < 0.001). No significant difference was detected between the SCH-CI group and the SCH-R group (p1 = 0.200).

Finally, the PNI values differed significantly across the groups (*p* = 0.006). The SCH-CI group demonstrated significantly lower PNI values than both the SCH-R (p1 = 0.008) and HC (p2 = 0.013) groups did, while no significant difference was observed between the SCH-R and HC groups (p3 = 1.000) (Table 3).

**Table 3:** Comparison of biochemical parameters between schizophrenia subgroups and healthy controls

Parameter	SCH-CI	SCH-*R*	HC	*p*-value	p1	p2	p3
Lymphocyte	1.97 ± 0.92 (a)	2.47 ± 0.90 (b)	2.34 ± 0.72 (ab)	0.043*	0.037	0.252	1
ALB	42.17 ± 4.65 (a)	43.71 ± 3.10 (ab)	44.43 ± 2.42 (b)	0.023*	0.142	0.019	0.804
CRP	4.20(2.18–8.38) (a)	3.65(1.60–5.73) (a)	1.10(0.50–3.72) (b)	< 0.001β	1.000	< 0.001	< 0.001
CRP/ALB ratio	0.09(0.05–0.19) (a)	0.09(0.04–0.14) (a)	0.03(0.01–0.08) (b)	< 0.001β	1.000	< 0.001	< 0.001
CALLY	0.16(0.06–0.34) (a)	0.26 (0.10–0.64) (a)	0.93(0.29–1.87) (b)	< 0.001β	0.200	< 0.001	< 0.001
PNI	52.02 ± 6.92 (a)	56.07 ± 5.88 (b)	56.11 ± 4.32 (b)	0.006*	0.008	0.013	1

^β^ Kruskal‒Wallis test, * ANOVA,

p1: SCH-CI vs. SCH-R

p2: SCH-CI vs. HC

p3: SCH-R vs. HC

**Fig. 1:**
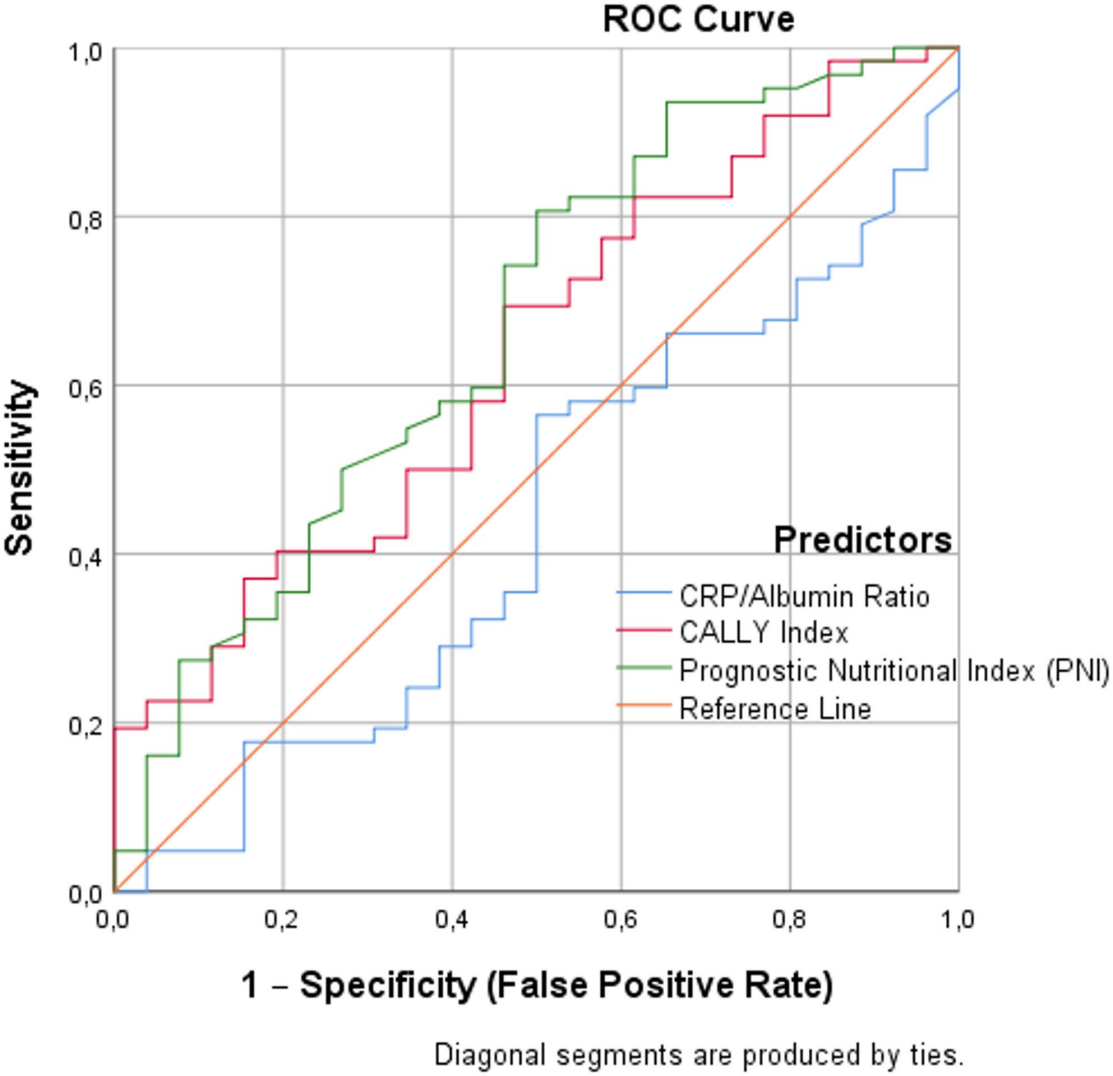
Receiver operating characteristic (ROC) curves of the PNI, CALLY index, and CRP/ALB ratio for discrimination of cognitive impairment among patients with schizophrenia (SCH-CI vs. SCH-R). The y-axis represents sensitivity and the x-axis represents 1 − specificity (false positive rate). The diagonal reference line indicates no-discrimination performance (AUC = 0.50)

**Table 4:** ROC analysis results for the CRP/ALB ratio, CALLY index, and PNI in distinguishing SCH-CI from SCH-R

	AUC (95% CI)	Std. Error^a^	*p*	Cut-off value	Sensitivity(95%CI) -Specificity(95%CI)(%)	Likelihood ratio
CRP/ALB ratio	0.435(0.303–0.566)	0.067	0.335			
CALLY index	0.636(0.511–0.760)	0.064	0.045	0.189	69.4(63.3–85.8))- 53.8(20.2–59.4)	1.50
PNI	0.670(0.543–0.797)	0.065	0.012	51.3	86.0(68.6–89.6)- 50.0 (29.9–70.1)	1.61

CRP/ALB C-reactive protein/albumin ratio, PNI Prognostic nutritional index, CALLY index C-reactive protein–albumin–lymphocyte index

ROC analyses were performed to evaluate the diagnostic performance of biochemical markers in distinguishing between the SCH-CI and SCH-R groups. The AUC value for the CRP/ALB ratio was 0.435 (95% CI: 0.303–0.566; *p* = 0.335), indicating that this marker did not significantly discriminate between the two schizophrenia subgroups.

In contrast, the CALLY index showed statistically significant discriminatory power, with an AUC of 0.636 (95% CI: 0.511–0.760; *p* = 0.045). A cut-off value of 0.189 yielded a sensitivity of 69.4% and a specificity of 53.8% (LR = 1.50), suggesting moderate performance in differentiating SCH-CI patients from SCH-R patients.

Similarly, the PNI demonstrated a significant diagnostic capacity, yielding an AUC of 0.670 (95% CI: 0.543–0.797; *p* = 0.012). At the cut-off value of 51.3, the sensitivity was 86.0%, and the specificity was 50.0% (LR = 1.61). These results suggest that the PNI showed modest discriminatory performance in distinguishing SCH-CI from SCH-R. (Table 4). (Fig. 1).

**Table 5:** Multivariable logistic regression analysis of the association between cognitive impairment and PNI, CALLY index, and CRP/ALB Ratio

Model	OR	95% CI	p*	Model χ² (df)	Nagelkerke *R*²	HL-test *p*
**Model 1**						
PNI	0.895	0.800–1.001	0.052	19.465 (8)	0.282	0.527
**Model 2**						
CALLY index	0.112	0.013–0.943	0.044	21.439 (8)	0.308	0.266
**Model 3**						
CRP/ALB ratio	13.363	0.147–1212.867	0.26	16.683 (8)	0.246	0.688

*Binary Logistic Regression; adjusted for age, sex, body mass index, smoking status, education year, depot antipsychotic use, and illness duration

To further account for potential confounding, multivariable logistic regression analyses were performed including age, sex, body mass index (BMI), smoking status, education year, depot antipsychotic use, and illness duration as covariates. In the model including the Prognostic Nutritional Index (PNI), lower PNI values showed a borderline association with cognitive impairment after adjustment (OR = 0.895; 95% CI: 0.800–1.001; *p* = 0.052; Nagelkerke R² = 0.282; Hosmer–Lemeshow *p* = 0.527). In the model including the CALLY index, lower CALLY values remained independently associated with cognitive impairment (OR = 0.112; 95% CI: 0.013–0.943; *p* = 0.044; Nagelkerke R² = 0.308; Hosmer–Lemeshow *p* = 0.266). By contrast, the CRP/albumin ratio was not associated with cognitive impairment in the adjusted model (OR = 13.363; 95% CI: 0.147–1212.867; *p* = 0.260; Nagelkerke R² = 0.246; Hosmer–Lemeshow *p* = 0.688). Overall, these findings suggest that, after further adjustment, the association was more robust for the CALLY index than for the PNI or the CRP/albumin ratio (Table 5).

## Discussion

This study demonstrated that cognitive impairment in patients with schizophrenia is associated with higher CRP/ALB ratios and lower PNI and CALLY index values. Specifically, patients with cognitive impairment presented lower albumin and lymphocyte levels and higher CRP levels, resulting in elevated CRP/ALB ratios and reduced PNI and CALLY indices. Cognitive function was evaluated using the Montreal Cognitive Assessment (MoCA), a screening instrument that has been validated in schizophrenia and is widely applied in clinical settings [16–18]. While the MoCA provides a practical and feasible assessment of global cognitive function, it does not allow detailed examination of domain-specific cognitive deficits, which are central to schizophrenia. Therefore, the present findings should be interpreted within the framework of global rather than domain-specific cognitive functioning.

Malnutrition, unhealthy lifestyle behaviors, metabolic side effects of antipsychotic medications, and chronic inflammation are frequently observed in individuals with schizophrenia [19]. These factors may adversely influence both overall health and cognitive performance. Consistent with previous findings, CRP levels were significantly higher in both schizophrenia groups than in the healthy control group, although no significant difference was observed between the SCH-CI and SCH-R groups. CRP is a well-established inflammatory marker [20], and elevated CRP levels have been widely reported in patients with schizophrenia [21]. Elevated CRP may directly influence central nervous system function by promoting microglial activation, increasing blood–brain barrier permeability, and modulating monoaminergic neurotransmission, possibly through mechanisms involving altered neurotransmission, neurodegeneration, immune response alterations, and oxidative stress, collectively contributing to synaptic dysfunction and reduced neuroplasticity that are strongly linked to cognitive decline in schizophrenia [22, 23].

In our study, albumin levels were significantly lower in the SCH-CI group than in the healthy control group; however, no significant difference was observed between the SCH-R and HC groups. This may be attributed to relatively better nutritional status and metabolic stability in schizophrenia patients with preserved cognitive function. Albumin is a classical marker of nutritional status and chronic disease burden; low levels may reflect malnutrition, chronic illness, or impaired protein synthesis [24]. Individuals with schizophrenia commonly experience nutritional imbalances, lifestyle-related problems, and medication side effects (e.g., increased metabolic syndrome risk), all of which may contribute to decreased albumin levels [25]. Albumin functions as a major antioxidant. Mechanistically, hypoalbuminemia can weaken systemic antioxidant defenses, exacerbating oxidative neuronal injury, impairing mitochondrial function and synaptic signaling, and increasing vulnerability within frontal and hippocampal circuits that support cognitive processes [26, 27]. Previous research has reported significantly lower total protein, albumin, and globulin levels in schizophrenia patients than in healthy individuals [28], supporting a role for inflammatory processes in reducing albumin synthesis. However, serum total protein and globulin levels were not available in the present dataset; therefore, we could not examine whether these broader protein-related markers showed patterns similar to those observed for albumin.

In our study, the CRP/ALB ratio was greater in both schizophrenia groups than in the healthy control group. To the best of our knowledge, no prior study has specifically examined the CRP/ALB ratio in the SCH-CI subgroup. The CRP/ALB ratio is considered more informative than CRP or albumin alone, as it reflects both systemic inflammation and nutritional depletion in patients with schizophrenia [15].

Most studies investigating the prognostic nutritional index (PNI) in psychiatric disorders have primarily focused on depressive disorders, whereas research on schizophrenia remains limited [29, 30]. In one study, PNI levels were reported to be significantly lower in patients with schizophrenia compared to healthy controls [31]. Another study including patients with both schizophrenia and bipolar disorder demonstrated reduced PNI levels in both patient groups relative to healthy controls, suggesting a shared immunonutritional impairment across these disorders [32]. In the present study, consistent with the existing literature, PNI levels were lower in both the SCH-CI and SCH-R groups compared to the HC group. However, to date, only one previous study has specifically examined the relationship between PNI and cognitive impairment in patients with schizophrenia. In line with our findings, that study demonstrated a negative association between PNI levels and cognitive dysfunction [33]. In multivariable analyses adjusting for relevant demographic and clinical covariates, the association between PNI and cognitive impairment was attenuated and became borderline after inclusion of education year and illness duration. Albumin and lymphocyte count may each be weakly associated with cognitive impairment in isolation but jointly capture a more comprehensive immunonutritional phenotype that single markers miss.

A low PNI reflects increased systemic inflammation and impaired immunonutritional status. Reduced albumin levels are associated with diminished antioxidant capacity, whereas decreased lymphocyte counts indicate a weakened immune response. Lymphopenia may further impair immune regulation of chronic neuroinflammatory states by altering cytokine balance and reducing adaptive immune surveillance. In schizophrenia, persistent low-grade inflammation combined with inadequate immune modulation may amplify microglial overactivation and synaptic pruning abnormalities, thereby worsening cognitive outcomes. Collectively, these alterations may be linked to processes such as neuroinflammation, increased oxidative stress, and disrupted synaptic function, which may contribute to cognitive impairment [34].

We also observed lower CALLY index values in both schizophrenia groups than in the control group. To our knowledge, this is the first study to explore the association between the CALLY index and cognitive impairment in patients with schizophrenia. A prior study in patients with type 2 diabetes reported lower CALLY values in individuals with cognitive impairment [35], supporting the notion that systemic inflammatory–nutritional imbalance may be linked to cognitive vulnerability across different chronic disorders. The CALLY index has been primarily investigated in non-psychiatric medical conditions and has been shown to reflect the combined effects of systemic inflammation, immune competence, and nutritional status [14]. Unlike single biomarkers, this composite index integrates CRP, albumin, and lymphocyte levels, thereby capturing multiple biological processes simultaneously. Such multidimensional markers may be particularly informative in complex disorders like schizophrenia, where inflammation, oxidative stress, and immune dysregulation are believed to interact in a chronic and subtle manner [36]. From a biological perspective, a reduced CALLY index may reflect heightened inflammatory activity (elevated CRP), diminished antioxidant and neuroprotective capacity (reduced albumin), and impaired immune regulation (lymphopenia) [14, 36–38]. These mechanisms are consistent with contemporary neurobiological models of schizophrenia, which emphasize the roles of neuroinflammation, oxidative stress, and immune alterations in the development of cognitive dysfunction [36]. Collectively, these processes may be associated with synaptic dysfunction, neuronal degeneration, and disturbances in neurotransmitter systems, potentially increasing the risk of cognitive decline in patients with schizophrenia. Although causal relationships cannot be established within the cross-sectional design of the present study, the observed associations suggest that the CALLY index may represent a clinically accessible surrogate marker of systemic processes potentially linked to cognitive impairment. In our multivariable analysis, the CALLY index remained independently associated with cognitive impairment after adjustment for demographic and clinical covariates, suggesting that this composite marker may capture inflammatory–nutritional vulnerability more robustly than single-component indicators under stricter adjustment.

Clinically, the PNI and CALLY indices are practical, inexpensive, and derived from routine laboratory tests, making them promising potential biomarkers for identifying individuals who may be at increased risk of cognitive impairment in patients with schizophrenia. However, the modest specificity values observed for PNI (50.0%) and CALLY (53.8%) indicate that these indices may have limited utility as standalone diagnostic tools. Their clinical applicability may be improved by integrating them into multimarker models that combine inflammatory, metabolic, and clinical variables. Accordingly, these indices may be more suitable for risk stratification or screening support than for definitive identification of cognitive impairment when used alone. Furthermore, external validation in independent cohorts is necessary to confirm the stability and generalizability of the proposed ROC-derived cut-off values (PNI = 51.3; CALLY = 0.189) across different populations and clinical stages of schizophrenia. Another point requiring caution is the marked male predominance in the healthy control group, which may have influenced biomarker and cognitive comparisons, since sex-related biological differences can affect inflammatory profiles, nutritional markers, and cognitive performance. Because antipsychotic treatment characteristics may also influence inflammatory status, metabolic parameters, and cognition, residual treatment-related confounding cannot be excluded in the present study.

In addition, future data collection should incorporate validated measures of dietary patterns, physical activity, and sleep quality to adjust for these modifiable lifestyle factors and further strengthen the robustness of the findings.

A notable strength of this study is its contribution to the limited literature on integrated inflammatory–nutritional biomarkers in schizophrenia. The combined evaluation of CRP/ALB, PNI, the CALLY index, and related neuroimmune parameters provides a novel and clinically relevant framework, particularly given the scarcity of data on PNI and the absence of prior studies examining the CALLY index in relation to cognitive impairment in schizophrenia.

## Limitations

This study has several limitations. First, its cross-sectional design precludes causal inference. Second, although the overall sample size met a priori power requirements, the relatively small SCH-CI subgroup (*n* = 26) may have reduced the stability and precision of regression estimates. Third, cognitive function was assessed using the Montreal Cognitive Assessment (MoCA), a screening tool that does not permit domain-specific evaluation, limiting conclusions regarding which cognitive domains are most strongly associated with inflammatory–nutritional biomarkers; future studies should employ schizophrenia-validated neuropsychological batteries(e.g., MATRICS Consensus Cognitive Battery). Fourth, standardized symptom severity scales were not used, and residual symptom burden cannot be excluded. Fifth, the single-center design (*n* = 131), marked gender imbalance in the healthy control group (90.7% male), and the predominance of medicated patients limit external validity and may introduce residual confounding. Sex differences may influence both inflammatory–nutritional biomarkers and cognitive performance; therefore, the marked overrepresentation of males in the control group may have affected between-group comparisons of CRP, albumin, lymphocyte-based indices, and MoCA scores. Although sex was included as a covariate in the multivariable models, residual confounding cannot be fully excluded, particularly given the small subgroup size. Accordingly, the present findings should be interpreted with caution and may not be fully generalizable to female patients with schizophrenia. Future multicenter studies including larger and sex-balanced control and patient groups are needed to clarify whether the observed associations are consistent across men and women. In addition, antipsychotic type (typical vs. atypical), daily dose, chlorpromazine-equivalent exposure, and treatment duration were not systematically available for covariate adjustment. Because very few patients were receiving typical antipsychotics, further adjustment according to antipsychotic class was not methodologically reliable. Therefore, residual treatment-related confounding cannot be excluded, and the ability to disentangle illness-specific from treatment-related effects remains limited.

## Conclusion

In conclusion, the CRP/ALB ratio, PNI, and CALLY index represent relevant biomarkers reflecting inflammatory and immunonutritional processes in schizophrenia. Although ROC analyses in the present study demonstrated modest sensitivity and specificity, both the PNI and the CALLY index showed potential utility in differentiating patients with cognitive impairment from those without. However, given their limited specificity, these indices should be interpreted as supportive or risk-stratification markers rather than as standalone diagnostic tools. Notably, after adjustment for major demographic and clinical covariates, the CALLY index remained independently associated with cognitive impairment, whereas the association for PNI was attenuated to borderline significance. Nevertheless, future studies with larger, multicenter, and prospective designs are warranted to validate these biomarkers and to more comprehensively assess their predictive value before they can be incorporated into routine clinical practice.

## Data Availability

The datasets used and/or analysed during the current study are available from the corresponding author on reasonable request.

## Abbreviations

CRP/ALB: C-reactive protein/albumin ratio
PN: I Prognostic nutritional index
CALLY index: C-reactive protein–albumin–lymphocyte index
MoCA: Montreal cognitive assessment scale
SCH-R: Schizophrenia patients with preserved cognitive function
SCH-CI: Schizophrenia group with cognitive impairment
HC: Healthy control
DSM-5-TR: Diagnostic and statistical manual of mental disorders, fifth edition, text revision
